# Development of hepatoma-derived, bidirectional oval-like cells as a model to study host interactions with hepatitis C virus during differentiation

**DOI:** 10.18632/oncotarget.19108

**Published:** 2017-07-08

**Authors:** Masahiko Ito, Suofeng Sun, Takasuke Fukuhara, Ryosuke Suzuki, Miho Tamai, Toyohiko Yamauchi, Kenji Nakashima, Yoh-ichi Tagawa, Shigetoshi Okazaki, Yoshiharu Matsuura, Takaji Wakita, Tetsuro Suzuki

**Affiliations:** ^1^ Department of Virology and Parasitology, Hamamatsu University School of Medicine, Shizuoka, Japan; ^2^ Department of Molecular Virology, Research Institute for Microbial Diseases, Osaka University, Osaka, Japan; ^3^ Department of Virology II, National Institute of Infectious Diseases, Tokyo, Japan; ^4^ School of Life Science and Technology, Tokyo Institute of Technology, Kanagawa, Japan; ^5^ Central Research Laboratory, Hamamatsu Photonics K.K., Shizuoka, Japan; ^6^ Department of Medical Spectroscopy, Hamamatsu University School of Medicine, Shizuoka, Japan

**Keywords:** hepatitis C virus, epigenetic reprogramming, hepatoma cell, oval cell, miR200a, Immunology and Microbiology Section, Immune response, Immunity

## Abstract

Directed differentiation of human stem cells including induced pluripotent stem cells into hepatic cells potentially leads to acquired susceptibility to hepatitis C virus (HCV) infection. However, cellular determinants that change their expression during cell reprogramming or hepatic differentiation and are pivotal for supporting the HCV life cycle remain unclear. In this study, by introducing a set of reprogramming factors, we established HuH-7-derived oval-like cell lines, Hdo-17 and -23, which possess features of bipotential liver precursors. Upon induction of hepatocyte differentiation, expression of mature hepatocyte markers and hepatoblast markers in cells increased and decreased, respectively. In contrast, in response to cholangiocytic differentiation induction, gene expression of epithelium markers increased and cells formed round cysts with a central luminal space. Hdo cells lost their susceptibility to HCV infection and viral RNA replication. Hepatic differentiation of Hdo cells potentially led to recovery of permissiveness to HCV RNA replication. Gene expression profiling showed that most host-cell factors known to be involved in the HCV life cycle, except CD81, are expressed in Hdo cells comparable to HuH-7 cells. HCV pseudoparticle infectivity was significantly but partially recovered by ectopic expression of CD81, suggesting possible involvement of additional unidentified factors in HCV entry. In addition, we identified miR200a-3p, which is highly expressed in Hdo cells and stem cells but poorly expressed in differentiated cells and mature hepatocytes, as a novel negative regulator of HCV replication. In conclusion, our results showed that epigenetic reprogramming of human hepatoma cells potentially changes their permissivity to HCV.

## INTRODUCTION

Approximately 130-200 million people are chronically infected with hepatitis C virus (HCV) worldwide [1]. HCV is a causative agent of chronic hepatitis, cirrhosis, and hepatocellular carcinoma. HCV is an enveloped, positive single-stranded RNA virus belonging to the genus hepacivirus in the family of *Flaviviridae*.

Among cell lines known to support complete propagation of HCV (see [2]), HuH-7, a well-differentiated hepatocellular carcinoma cell line, has been most commonly used for HCV research. Human and macaque induced pluripotent stem (iPS) and embryonic stem (ES) cells have also been used for HCV studies. Although it has been demonstrated that hepatic differentiation is closely related to susceptibility of iPS/ES cells to HCV infection and propagation [3–7], it seems that their susceptibility to HCV is limited and clearly less compared to HuH-7 cells. Further, cellular determinants that change their expression during reprogramming or hepatic differentiation from iPS/ES cells and are pivotal for supporting the HCV life cycle are still unknown.

Several reports have shown that iPS-like cells were potentially established by introducing reprogramming factors into cancer cells according to pluripotent marker expression [8–11]. In some cases, induction of differentiation from these iPS-like cells led to changes in cell proliferation, tumorigenesis, and/or chemosensitivity.

In this study, to assess the effect of epigenetic modification on susceptibility to HCV and to identify host cell factors involved in regulation of the HCV life cycle in a cell differentiation-dependent manner, we introduced a set of reprogramming factors into HuH-7 cells and established cell lines possessing oval cell features, termed HuH-7-derived oval-like (Hdo). We found that Hdo cells lost their susceptibility to HCV infection and replication but maintained support to propagate Japanese encephalitis virus (JEV), another *Flaviviridae* member, and hepatitis B virus (HBV), another hepatotropic virus. Based on comparative analyses of gene expression profiles between Hdo and HuH-7 cells, miR200a-3p that is highly expressed in Hdo cells and poorly-differentiated cells was identified as a host factor that negatively regulates HCV replication.

## RESULTS

### Generation and characterization of Hdo cells

**Figure 1:**
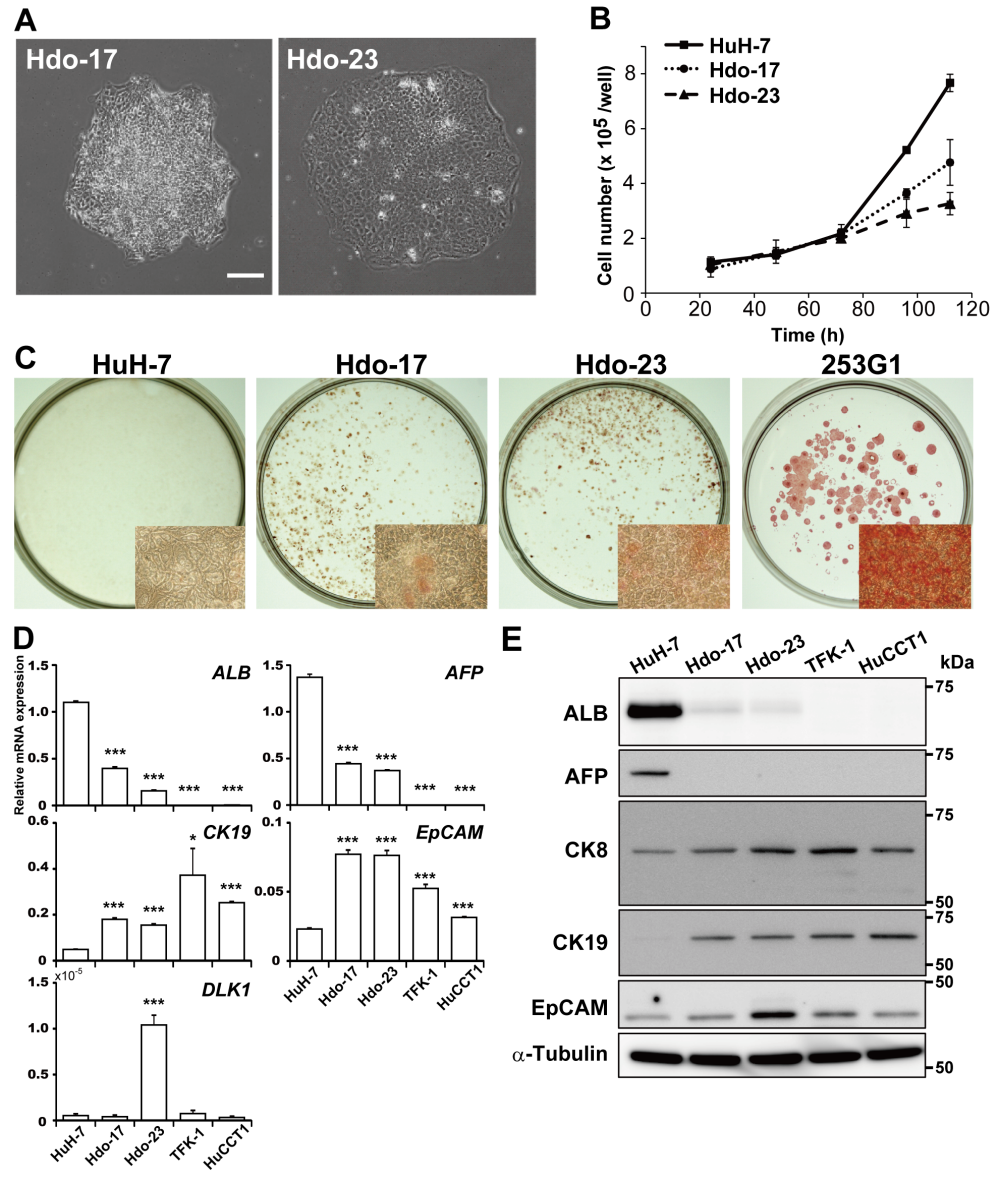
Generation and characterization of Hdo cells **A**. HuH-7 cells were infected with a retrovirus expressing *OCT3/4*, *SOX2*, *KLF4*, *LIN28*, and *NANOG* genes. Two cell clones (Hdo-17 and -23) were obtained after 40 days of culture. Bar indicates 200 μm. **B**. Cell growth was measured by counting cell numbers after plating of 1×10^5^ cells/well in 24-well plates. **C**. ALP expression in HuH-7, Hdo-17, Hdo-23, and 253G1 cells was examined by staining with the Leukocyte Alkaline Phosphatase kit at 3 days after passage. Inset: higher magnification (6× objective). **D**. and **E**. At 5 days after passage, total RNA and protein in HuH-7, Hdo-17, Hdo-23, TFK-1, and HuCCT1 cells were extracted. Expression of liver markers was measured by qRT-PCR (D) and Immunoblotting (E). Data were normalized to the expression of *GAPDH* mRNA. (B)-(E) Assays were performed in triplicate. (B) and (D) Results are presented as means ± SEM (*n* = 3). Statistically significant differences compared with HuH-7 cells are shown. *p < 0.05, **p < 0.01, ***p < 0.001, Student's *t*-test.

To generate undifferentiated cells derived from the HuH-7 cell line, which exhibits high susceptibility to HCV infection, cell reprogramming was induced via transduction with retroviral vectors expressing *OCT3/4*, *SOX2*, *KLF4*, *LIN28*, and *NANOG* genes, which are essential for establishment and maintenance of the pluripotent state. Newly generated cell colonies were identified on day 40 post-transduction according to typical pluripotent colony morphology. After expansion of cells, two lines of reprogrammed cells (termed Hdo-17 and -23) were established (Figure 1A). Hdo cells underwent a high rate of apoptosis after passaging of single cells similar to iPS cells (data not shown). Calculated doubling times of Hdo-17 and -23 cells (36 h and 51 h, respectively) were longer than that of HuH-7 cells (25 h) (Figure 1B). Similar results were obtained by ATP quantitation (Supplementary Figure 1A). Although the undifferentiated state of ES and iPS cells can be characterized by a high level of ALP expression, Hdo cells exhibited moderate ALP activity, lower than that of human iPS cell line, 253G1 (Figure 1C) [12]. Among pluripotency markers, expression of *OCT3/4*, *KLF4*, *LIN28*, and *NANOG* mRNAs in Hdo cells were markedly higher than that in HuH-7 cells. Expression of *SOX2* and *REX1* mRNAs was not observed in Hdo cells similar to HuH-7 cells (Supplementary Figure 1B). Immunofluorescence staining using antibodies against the pluripotency surface markers showed that expression of SSEA-1 was detectable in Hdo cells but TRA1-81, TRA-1-60, SSEA-3, and SSEA-4 were not (data not shown). Notably, mRNA expression of *ALB* and *AFP*, markers of mature hepatocytes and hepatoblasts, respectively, was significantly reduced (*p* < 0.001) but expression of cholangiocyte and oval-cell markers *EpCAM* and *CK19* was induced in Hdo cells (Figure 1D). The expression of DLK1, which is considered as a marker for fetal hepatic stem/progenitor cells, was observed in Hdo-23. Differential expression of these markers was also observed at the protein level (Figure 1E; Supplementary Figure 1C). In contrast, expression of liver-specific genes such as *CK8*, *TTR*, *TAT*, *HNF4A*, *CYP3A4*, and *AAT* was maintained in Hdo cells as well as HuH-7 cells (Figure 1E; Supplementary Figure 1D). Glycogen storage of Hdo cells as detected by PAS staining was found to be largely comparable to that in HuH-7 cells (Supplementary Figure 1E).

Collectively, it is likely that Hdo cells derived from HuH-7 cells are not completely reprogrammed into stem cells but have distinct features seen in hepatic oval cells according to the expression pattern of liver development markers, e.g., ALB+, AFP+, CK19+, and EpCAM+.

### Bidirectional differentiation of Hdo cells

**Figure 2:**
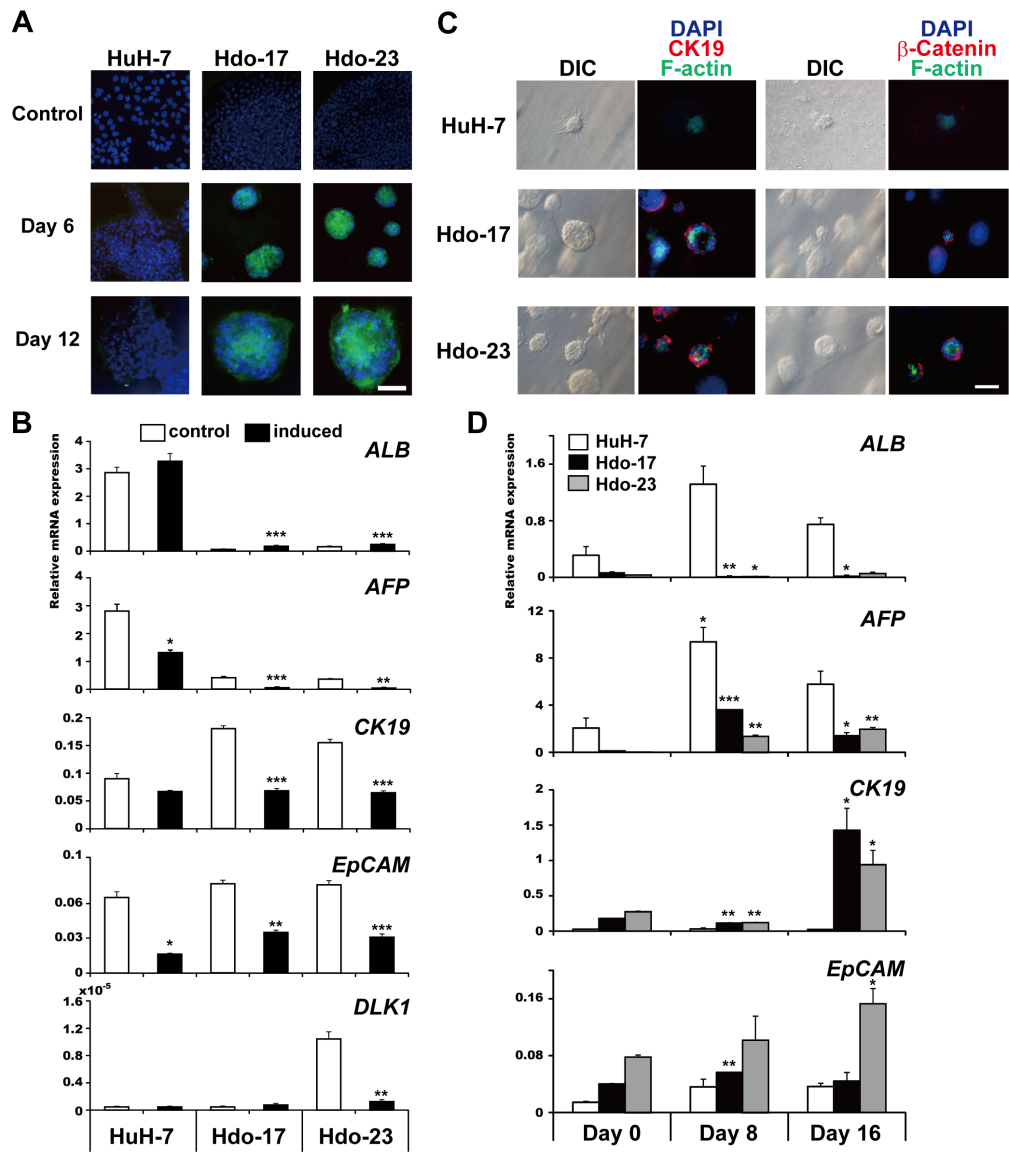
Bidirectional differentiation potential of Hdo cells **A**. HuH-7, Hdo-17, and Hdo-23 cells were cultured in hepatic differentiation medium for 6 and 12 days and stained with anti-albumin antibody (green) and DAPI (blue). Bar indicates 100 μm. **B**. After 12 days of culture in hepatic induction medium (black bars) or control medium (white bars), total RNA from HuH-7, Hdo-17, and Hdo-23 cells was isolated and the expression of liver markers was measured by qRT-PCR. Data are normalized to the expression of *GAPDH* mRNA. **C**. HuH-7, Hdo-17, and Hdo-23 cells were cultured in cholangiocytic induction medium for 12 days and stained with Alexa Fluor 594 anti-CK19 antibody or anti-β-catenin antibody (red). F-actin expression was determined by staining with Alexa Fluor 488-conjugated phalloidin (green). Nuclei were stained with DAPI (blue). Bar indicates 50 μm. **D**. At 0, 8, and 16 days after culture in cholangiocytic induction medium, total RNAs of HuH-7 (white bars), Hdo-17 (black bars), and Hdo-23 (grey bars) cells were isolated and the expression of liver markers was measured by qRT-PCR. Data were normalized to the expression of *GAPDH* mRNA. All assays were performed in triplicate. (B) and (D) Results are presented as means ± SEM (*n* = 3). Statistically significant differences compared with control cells with no induction treatment (B) or cells at Day 0 (D) are shown. *p < 0.05, **p < 0.01, ***p < 0.001, Student's *t*-test.

Oval cells, which express markers of both fetal hepatocytes and biliary cells, are known to have the capacity to differentiate into hepatocytes and bile duct cells during liver regeneration [13, 14]. To test whether Hdo cells are able to exhibit oval cell response, we first evaluated hepatic differentiation potential by culturing cells plated on type I collagen in hepatocyte differentiation medium. Under this culture condition, Hdo cells but not HuH-7 cells clonally proliferated and formed a colony (Figure 2A). By quantitative phase imaging, the height of Hdo cells was higher than that of HuH-7 cells but formed a flattened shape similar to HuH-7 cells after hepatic induction (Supplementary Figure 2A, 2B). After 6 days of Hdo cell culture, *ALB* expression was induced, whereas *AFP* expression was decreased (Figure 2A, 2B). Under hepatic induction, mRNA expression of *EpCAM* and *CK19* was decreased (Figure 2B). Thus, Hdo cells cultured in hepatic induction medium can be differentiated into hepatic lineage cells.

To determine cholangiocytic differentiation potential, Hdo cells were embedded in a Matrigel/type I collagen mixture and cultured with cholangiocytic differentiation medium. After 12 days of culture, large cyst-like structures consisted of cells wrapped around the lumen were generated (Figure 2C). Immunofluorescence analysis revealed that CK19 and β-catenin were expressed outside of these cysts and F-actin, a marker of the apical domain, was expressed around the luminal space. This finding indicates cysts generated in culture have cellular polarity (Figure 2C), suggesting that Hdo cells have the potential to form bile duct-like structures. HuH-7 cells did not produce the luminal structure with polarity under this culture condition. As expected, mRNA expression of *CK19* and *EpCAM* was significantly elevated in response to cholangiocytic differentiation induction (*p* < 0.01) (Figure 2D). Although induced expression of *AFP* mRNA was observed in Hdo cells, the levels were consistently lower than those in induced HuH-7 cells. mRNA expression of *ALB* was not detected in Hdo cells under this culture condition. Taken together, the findings demonstrate that Hdo cells indeed have the ability to bidirectionally differentiate into hepatocytes and bile duct cells in cell culture. Thus, the name of the cell lines, Hdo, originated from “HuH-7-derived oval-like.”

### Impaired susceptibility of Hdo cells to HCV entry

**Figure 3:**
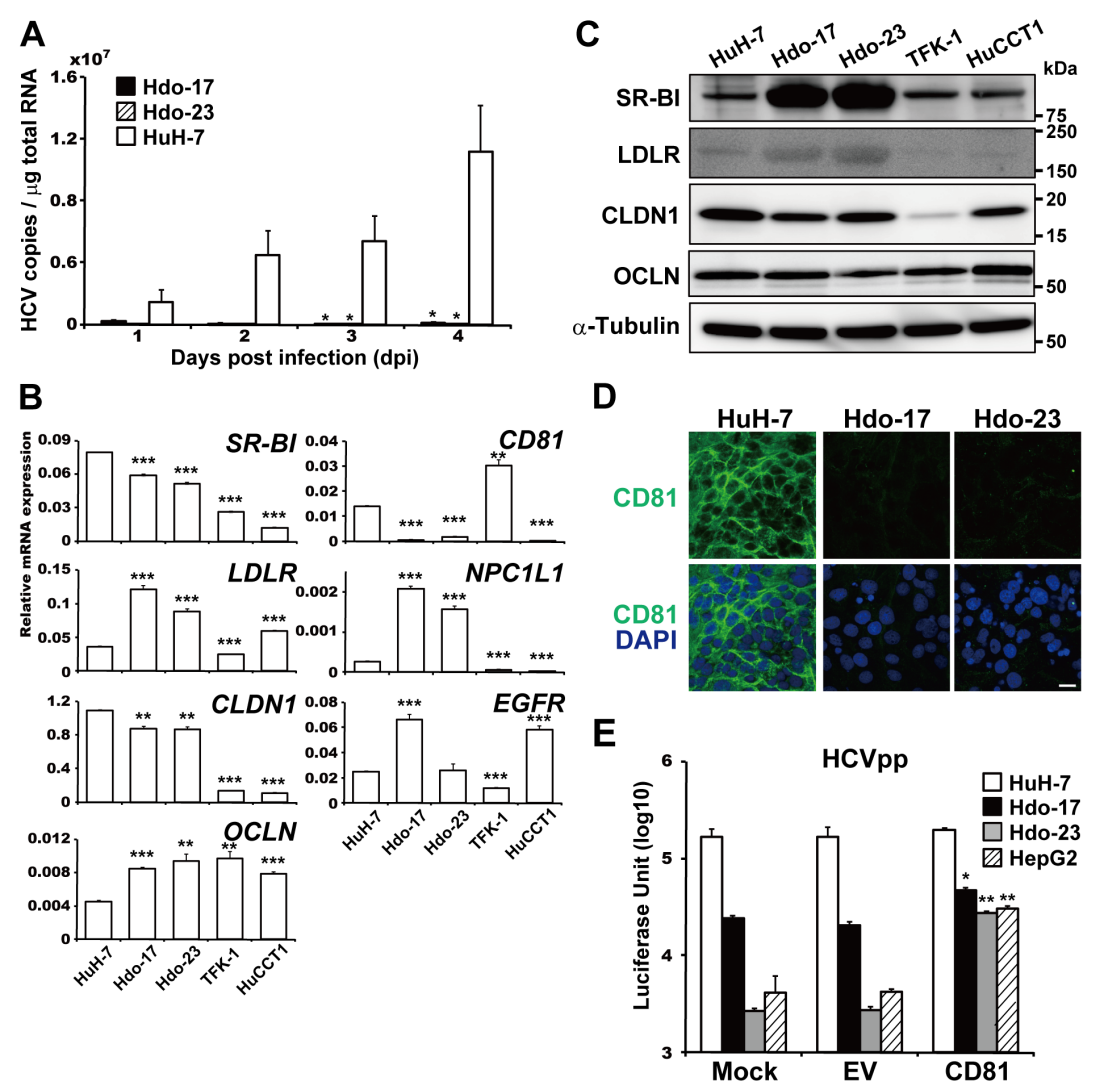
Impaired susceptibility of Hdo cells to HCV entry **A**. Hdo-17 (black bars), Hdo-23 (cross-hatched bars), and HuH-7 (white bars) cells were infected with JFH-1 derived HCVcc (MOI=1). HCV RNA copies in cells were measured by qRT-PCR. Values were normalized to total RNA concentration in cell lysates. **B**. and **C**. Expression of HCV receptors in HuH-7, Hdo-17, Hdo-23, TFK-1, and HuCCT1 cells was measured by qRT-PCR (B) and immunoblotting (C). Data were normalized to the expression of *GAPDH* mRNA. **D**. Expression of cell surface CD81 was examined by immunostaining with anti-CD81 antibody (green) and DAPI (blue). Upper and lower panels show CD81 expression and merged images with DAPI, respectively. Bar indicates 20 μm. **E**. One day after transfection with pcDNA3.1 (empty vector, EV) or pcDNA-CD81, HuH-7 (white bars), Hdo-17 (black bars), Hdo-23 (grey bars), and HepG2 (cross-hatched bars) cells were infected with HCVpp. NanoLuc activity was measured at 1 day post-infection. Data are expressed as means ± SEM (*n* = 3). All assays were performed in triplicate. (A), (C), and (E) Results are presented as means ± SEM (*n* = 3). Statistically significant differences compared with control cells [HuH-7 cells, (A) and (B)] or EV (E) are shown. *p < 0.05, **p < 0.01, ***p < 0.001, Student's *t*-test.

Cellular determinants that change expression during hepatic differentiation and/or dedifferentiation and are pivotal for regulating the HCV life cycle are still unclear. To investigate whether dedifferentiation of hepatic cells into progenitor cells with the oval cell phenotype can influence HCV susceptibility, Hdo and HuH-7 cells were infected with HCVcc, and viral RNA copies were measured by qRT-PCR. HCV RNA was not detectable in Hdo cells until 4 days post-infection, indicating that Hdo cells were not susceptible to HCV infection (Figure 3A). cDNA microarray, qRT-PCR, and immunoblotting to compare expression levels of possible key factors for HCV entry in Hdo cells with those in HuH-7 cells showed that levels of most factors such as SR-BI, LDLR, CLDN1, OCLN, NPC1L1, and EGFR in Hdo cells were comparable to or even higher than those in HuH-7 (Figure 3B, 3C; Supplementary Figure 3A, 3B). However, mRNA expression of *CD81* in Hdo cells was quite low (Figure 3B; Supplementary Figure 3A) and CD81 protein was not detectable in Hdo cells by immunostaining (Figure 3D). CD81 expression on the surface of Hdo cells was lower than 1% of HuH-7 cells as determined by flow cytometry (Supplementary Figure 3C). Considering the hypothesis that loss of CD81 expression could result in impaired susceptibility of the cells to HCV entry, it may be likely that ectopic expression of CD81 in Hdo cells can rescue viral entry into the cells. To address this, infectivity with HCVpp, an HIV-1-based pseudotype virus bearing HCV envelope proteins, in Hdo cells with or without transfection with the CD81-expession vector was evaluated. While HCVpp infectivity in Hdo cells was approximately 7- and 60-fold lower in Hdo-17 and -23 cells, respectively, compared to that in HuH-7 cells, ectopic expression of CD81 (Supplementary Figure 3C) led to considerable recovery of infectivity (Figure 3E). However, increased infectivity obtained by ectopic CD81 expression in Hdo cells remained significantly lower than that in HuH-7 cells (*p* < 0.001). These results strongly suggest that, although loss of CD81 expression is a major cause of impaired susceptibility to HCV entry, change in expression of additional, unidentified key factor(s) involved in viral entry during dedifferentiation of HuH-7 cells potentially contributes to their distinct phenotype.

### Susceptibility of Hdo cells and cells with directed differentiation to HCV RNA replication

**Figure 4:**
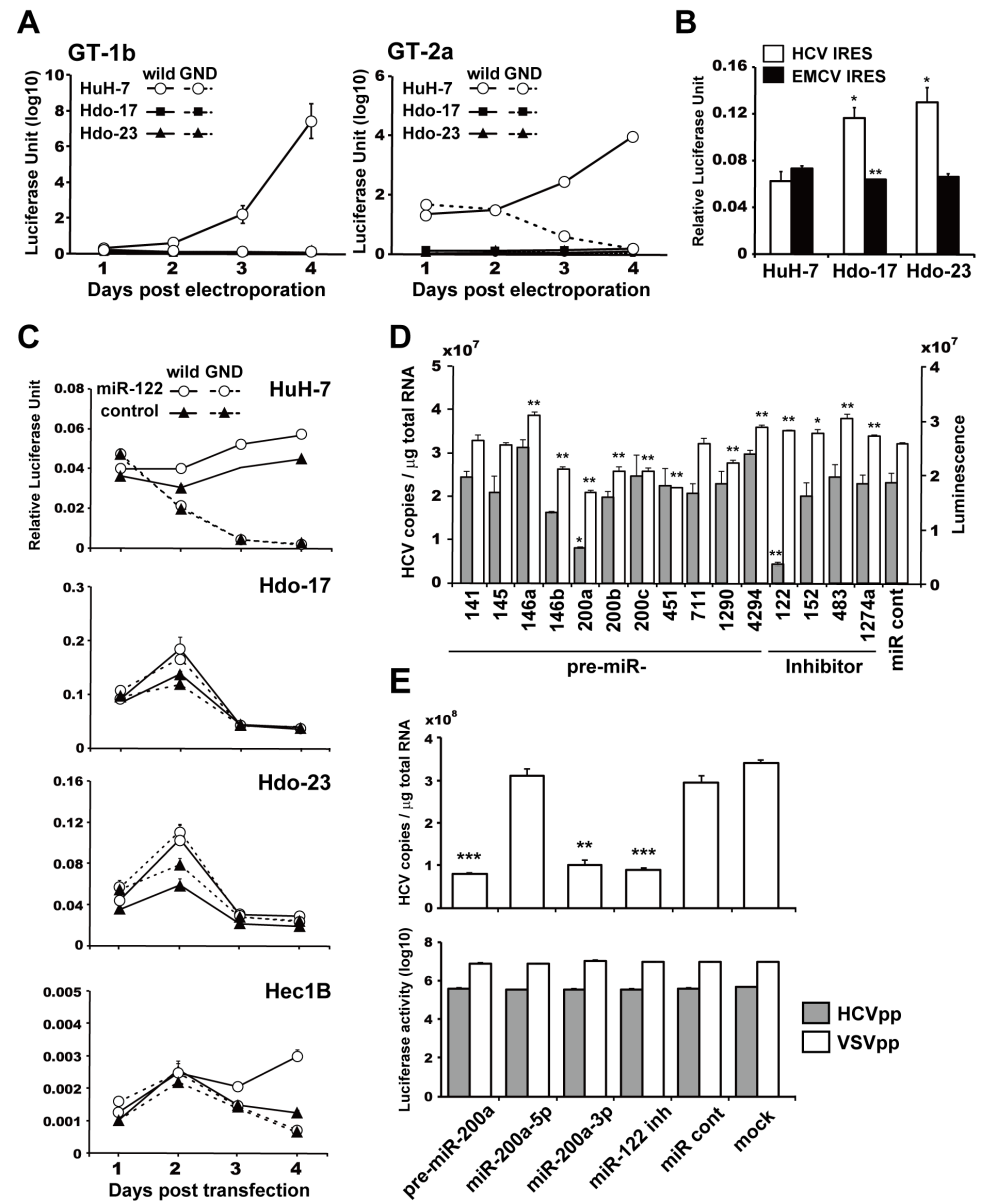
Susceptibility of Hdo cells to HCV RNA replication and involvement of miR-200a in replication **A**. HuH-7 (white circles), Hdo-17 (black squares), and Hdo-23 (black triangles) cells were transfected with subgenomic luciferase reporter replicon RNAs derived from HCV genotypes 2a [JFH-1, SGR-JFH1/GLuc wild (solid lines) and GND (dashed lines)] in left panel and 1b [Con1, SGR-Con1/GLuc wild (solid lines) and GND (dashed lines)] in right panel. Luciferase activities in supernatants were measured by the BioLux *Gaussia* Luciferase Assay Kit at the indicated time points. **B**. HuH-7, Hdo-17, and Hdo-23 cells were transfected with pRLucHCVLuc (white bars) or pRLucEMCVLuc (black bars). At 60 h after transfection, luciferase activity in cell lysates was measured by the dual-luciferase reporter assay system. The vertical axis indicates activity of firefly luciferase to that of *Renilla* luciferase. **C**. HuH-7, Hdo-17, Hdo-23, and Hec1B cells were infected with a lentivirus encoding miR-122 (white circles) or AcGFP as control (black triangles). Cells were transfected with pHH/SGR-JFH1/GLuc (solid lines) or pHH/SGR-JFH1/GLuc/GND (dashed lines) and pSV40-CLuc 48 h post-infection. Luciferase activity in the supernatant was measured by the BioLux Dual Luciferase Starter Kit at the indicated time points. Relative expression levels of secreted GLuc to CLuc in the supernatants are shown. **D**. HuH 5-15 cells, which are HCV genotype 1b-replicon cells, were transfected with miRNAs, miRNA inhibitors, and non-target miRNA control (miR cont) by ScreenFect A. At 3 days after transfection, HCV copies in cells were measured by qRT-PCR (grey bars). Cell viability was determined by the ATP concentration in cell lysates with the CellTiter-Glo Luminescent Cell Viability Assay (white bars). **E**. Huh7.5.1 cells were transfected with miRNAs, miR-122 inhibitor, or miR cont by the Xfect miRNA Transfection Kit. At 1 day after transfection, cells were infected with HCVcc (J6/JFH1, MOI=0.5). HCV copies in cells were measured by qRT-PCR at 3 days post-infection (upper graph). Huh7.5.1 cells were transfected with miRNAs, miR-122 inhibitor, or miR cont by the Xfect miRNA Transfection Kit. At 1 day after transfection, cells were infected with HCVpp (grey bars) or VSVpp (white bars). NanoLuc activity was measured at 1 day post-infection (lower graph). All assays were performed in triplicate. Results are presented as means ± SEM (*n* = 3). Statistically significant differences compared with HuH-7 cells (B) or miR cont (D) and (E) are shown. *p < 0.05, **p < 0.01, ***p < 0.001, Student's *t*-test.

Next, to examine susceptibility of Hdo cells to viral RNA replication, subgenomic luciferase reporter replicon RNAs derived from HCV genotypes 2a (JFH-1) and 1b (Con1) were introduced into Hdo cells and HuH-7 cells, and culture supernatants were collected at different time points. The reporter activities from Hdo cells were markedly lower than those from HuH-7 cells and were similar to that in cells transfected with a replication-defective mutant replicon (GND), indicating Hdo cells have no or little susceptibility to HCV RNA replication (Figure 4A). Translation activity mediated by HCV IRES was maintained or even higher in Hdo cells as judged by the bicistronic reporter assay (Figure 4B). One possible explanation for this finding may be that change in expression of host factor(s) important for HCV replication during dedifferentiation of HuH-7 cells results in loss of susceptibility to viral replication. Therefore, we performed cDNA microarray and qRT-PCR analyses to compare expression of genes that have been reported as host factors involved in HCV replication in Hdo-17 and -23 cells *versus* HuH-7 cells (Supplementary Figure 4A, Supplementary Table 1). Although expression levels of some factors in Hdo cells were moderately (30-60%) lower than those in HuH-7 cells, the levels of most host factors expressed in Hdo cells were comparable to those in HuH-7 cells. Expression of miR-122, a liver-specific microRNA that facilitates HCV genome amplification by stimulating RNA synthesis and promoting viral translation [15, 16], in Hdo cells was 10-20% lower than that in HuH-7 cells (Supplementary Figure 4B, bottom). To evaluate the effect of ectopic expression of miR-122 in Hdo cells on HCV replication, cells were transduced with a lentiviral vector encoding pri-miR-122, followed by transfection with an HCV subgenomic replicon (Figure 4C). While viral replication was increased in Hec1B cells, which are known to support replication when miR-122 is exogenously expressed [17], HCV replication was not observed in Hdo cells. Taken together, certain unidentified factor(s) whose expression changed during the reprogramming process are likely involved in loss of supporting HCV replication in Hdo cells.

miRNA microarray analysis further demonstrated that 11 miRNAs were upregulated and 3 miRNAs were downregulated (the threshold was set as two-fold) in both Hdo cell types compared to HuH-7 cells (Supplementary Figure 4B). To determine the possible involvement of these miRNAs or miRNA inhibitors in HCV replication, each pre-miRNA was introduced into subgenomic HCV replicon cells and viral RNA copies were determined. As shown in Figure 4D, transfection with miR-200a resulted in marked reduction of the viral RNA level. miR-200a is a known tumor suppressor miRNA that inhibits epithelial-mesenchymal transition and the initiating step of metastasis; it is also involved in differentiation, modulation of cell division, and apoptosis [18]. The expression of miR-200a was high in hepatoblasts but markedly low in mature hepatocytes (Supplementary Figure 5A). Analyses to address whether miR-200a is involved in regulation of the HCV life cycle were carried out as follows. First, reporter constructs carrying sequences of miRNA target sites or respective mutants downstream of the Luc gene were used to quantitatively evaluate the activities of pre-miR-200a, miR200a-5p, and miR200a-3p. While pre-miR200a suppressed the expression of genes containing a -5p or -3p target site, presumably at the post-transcriptional level, miR200a-5p and miR200a-3p only suppressed expression of genes with -5p and -3p target sites, respectively (Supplementary Figure 5B). Next, synthetic pre-miR-200a, miR200a-5p, or miR200a-3p was introduced into virus-infected cells (Figure 4E, upper) and replicon cells (Supplementary Figure 5C). We found that HCVcc propagation and replication of the viral subgenome were significantly impaired in HuH-7 cells treated with pre-miR200a or miR200a-3p but not in cells treated with miR200a-5p. Infectivity to HCVpp and VSVpp was not affected by the treatment (Figure 4E, lower), suggesting that miR200a-3p is potentially involved in HCV replication.

**Figure 5:**
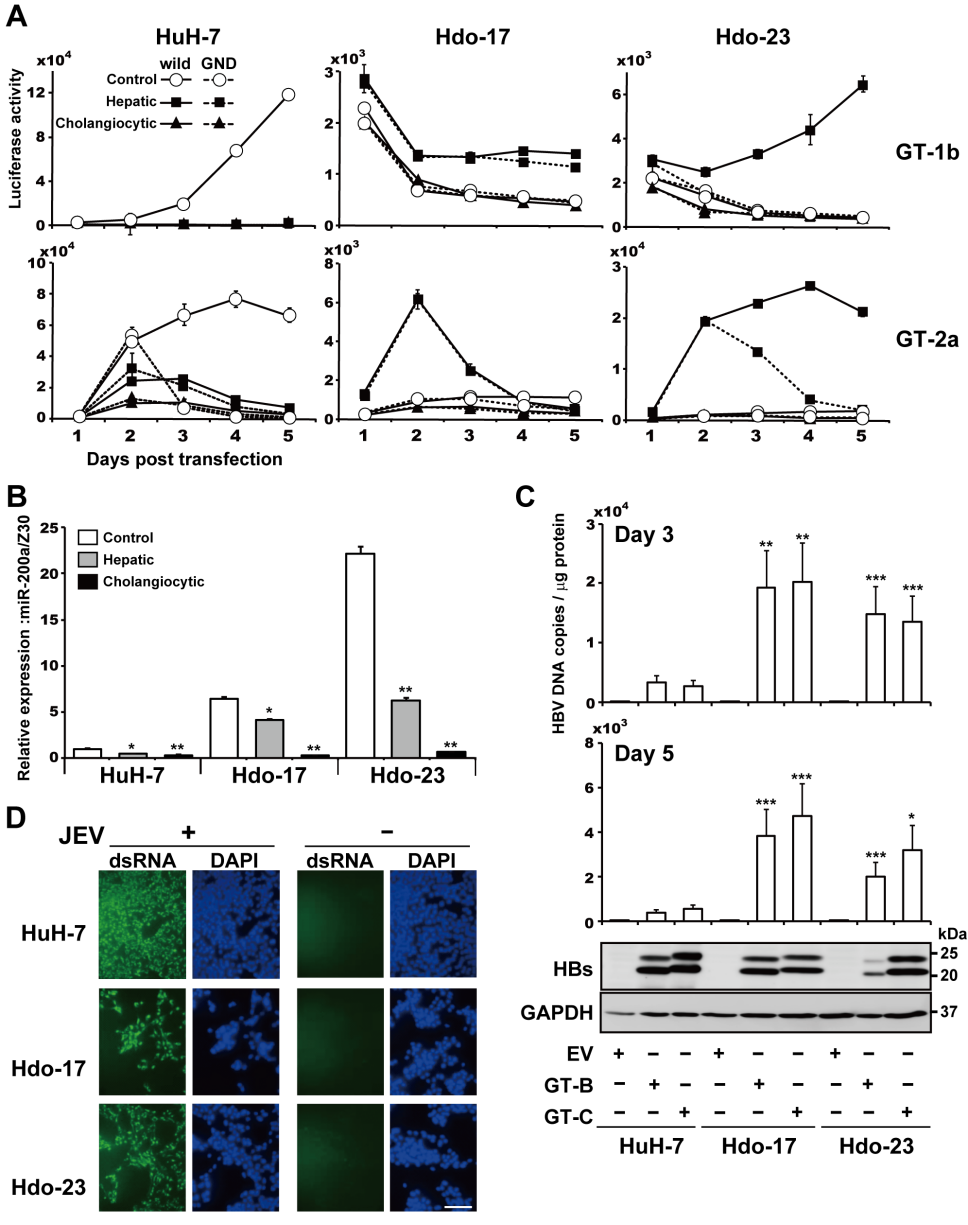
Susceptibility of directed differentiated cells from Hdo cells to HCV, HBV, and JEV replication **A**. Control, non-induced cells (white circles), hepatic induction cells (black squares), and cholangiocytic induction cells (black triangles) were transfected with subgenomic luciferase reporter replicon RNAs derived from HCV genotypes 1b [Con1, SGR-Con1/GLuc (solid lines) and GND (dashed lines)] in upper graphs and 2a [JFH-1, SGR-JFH1/GLuc (solid lines) and GND (dashed lines)] in lower graphs. Luciferase activities in supernatants were measured by the BioLux *Gaussia* Luciferase Assay Kit at the indicated time points. **B**. Expression of miR-200a in HuH-7, Hdo-17, Hdo-23, and their respective hepatic induction cells and cholangiocytic induction cells were examined by qRT-PCR using the TaqMan microRNA Reverse Transcription Kit with miR-200a specific RT primer from the TaqMan microRNA Assay. Expression of miR-200a was normalized to that of Z30 snoRNA (SNORD7), and relative expression levels to HuH-7 cells with no induction treatment are shown. **C**. HuH-7, Hdo-17, and Hdo-23 cells were transfected with pUC19, pUC-HBV-Bj (GT-B), or -Ce (GT-C). At 3 days or 5 days after transfection, particle-associated HBV DNAs in culture supernatants of transfected cells were measured by qRT-PCR (upper panel). Cell lysates were subjected to immunoblotting of HBs antigens and GAPDH (lower panel). **D**. HuH-7, Hdo-17, and Hdo-23 cells were infected with JEV (MOI=0.01). Cells were fixed and stained with dsRNA antibody (green) and DAPI (blue) at 3 days post-infection. Bar indicates 200 μm. All assays were performed in triplicate. (A)-(C) Results are presented as means ± SEM (*n* = 3). Statistically significant differences compared with control cells with no induction treatment (B) or HuH-7 cells (C) are shown. *p < 0.05, **p < 0.01, ***p < 0.001, Student's *t*-test.

We further examined whether induced differentiation of Hdo cells into hepatocytes or cholangiocytes confers susceptibility to HCV replication. After induction, Hdo cells were transfected with subgenomic reporter replicon RNAs derived from HCV genotype 1b (SGR-I389/GLuc) or 2a (SGR-JFH1/GLuc), and luciferase activities were evaluated. Interestingly, replication of viral RNAs was observed in hepatic-induced but not cholangiocytic-induced cells derived from Hdo-23 cells (Figure 5A), suggesting that differentiation of oval-like cells of hepatocyte lineage but not cholangiocyte lineage potentially leads to supporting HCV replication. In contrast, somewhat unexpectedly, hepatic induction of Hdo-17 cells did not result in acquired susceptibility to viral replication. It is generally accepted that factor-mediated direct reprogramming is stochastic and a small fraction of somatic cells that receive reprogramming factors attain pluripotent features [19, 20]. Although Hdo-17 and -23 cells possess comparable potential to induce hepatic differentiation as shown above (Figure 2A), molecular events such as epigenetic modifications may not occur uniformly during direct reprogramming of HuH-7 cells. As a result, some portion, but not all, of Hdo cells maintained the potential to support HCV replication. In hepatic differentiated Hdo cells, expression of miR-200a was lower than that in non-differentiated cells (Figure 5B).

### Hdo cells maintain susceptibility to HBV and JEV

HuH-7 cells support the replication of various human viruses including HBV and JEV. To investigate if susceptibility to HBV is also impaired in Hdo cells, cells were transfected with plasmids containing the 1.24-fold HBV genome derived from HBV genotypes B and C. Viral proteins in cells were determined by western blotting and particle-associated HBV DNA in culture supernatants were measured by qPCR (Figure 5C). The HBs protein level in Hdo-17 cells transfected with HBV genotype B or C and that in Hdo-23 cells transfected with genotype C seemed moderately lower than those in transfected HuH-7 cells. Compared to them, a decrease in the HBs level in Hdo-23 transfected with genotype B was more evident. However, viral DNA levels in the supernatants of Hdo cultures were 3- to 5-fold higher than those from HuH-7 cells. Thus, it appears that, unlike HCV, Hdo cells maintain susceptibility to HBV production. Further, the permissiveness of cells to JEV infection was tested (Figure 5D). Immunostaining indicated that efficiency of JEV infection and replication was comparable in Hdo and HuH-7 cells. Thus, impaired susceptibility to infection and replication during directed reprogramming of HuH-7 cells appears to be specific to particular virus(es).

## DISCUSSION

In this study, we established oval-cell-like, bipotent progenitor Hdo cells by introducing a set of reprogramming factors into HuH-7 cells. The cell clones (Hdo-17 and -23) have the capacity for unlimited proliferation and bidirectional differentiation into hepatocytes and cholangiocytes. Upon induction of directed differentiation, Hdo-derived cells exhibited gene expression profiles typically observed in mature hepatocytes and cholangiocytes, respectively (Figure 2B, 2D). Hdo cells also developed cyst-like morphological changes during cholangiocyte differentiation (Figure 2C). Previous studies have demonstrated that hepatic progenitor cells, which have characteristic features of oval cells, can be isolated from rodent livers [21–25]. Although it has long been thought that the oval cell counterpart in rodents exists in human liver, human progenitor cells characterized as oval cells have not yet been established. HepaRG cells, which were established from a liver tumor associated with HCV infection, exhibited clear morphological heterogeneity of both hepatocyte-like and biliary-like epithelial phenotypes [26]. However, it is unclear whether cells are capable of differentiating bidirectionally into hepatocytes and bile duct cells. While cells possessing bipotential differentiation characteristics were isolated from adult human liver, these cells were primarily cultured and markers specific for hepatic progenitor cells remained to be elucidated [27]. To our knowledge, this is the first report on the establishment of a human cell line possessing features of oval cells with an unlimited proliferative capacity.

Studies have shown that functional hepatocyte-like cells can be generated from ES cells or iPS cells [28]. However, their bipotential differentiation potency at the step of hepatic progenitor or oval-cell stage has not yet been demonstrated. To study the mechanisms of hepatocytic and/or cholangiocytic differentiation during liver development, Hdo cells may be a useful tool as a model of human liver progenitor cells. Another human hepatoma-derived cell line, HepG2, was also used for reprogramming mediated by Yamanaka factors in the same manner as HuH-7 cells. Judging from morphological analysis, ALP staining, and expression of pluripotency markers (data not shown), HepG2-derived reprogramed cells likely possess stem cell-like features rather than oval cell-like features. Alterations in cellular states caused by reprogramming factors may be attributable to differences in genetic and epigenetic features between HuH-7 and HepG2 cells.

We demonstrated here that permissiveness of HuH-7 cells to HCV infection and propagation can be lost via adding reprogramming factors, suggesting the involvement of epigenetic modification in regulation of gene expression important for several steps in the HCV life cycle. Although a prior study showed that iPS cells and iPS-derived definitive endoderm are not permissive to HCV infection [3], this study is the first to evaluate the potential susceptibility of cells with oval-cell-like features to HCV infection and replication. Our results indicated that loss of CD81 expression is a major cause of impaired susceptibility to HCV entry (Figure 3D, 3E). It may be likely, however, that dedifferentiation of HuH-7 cells resulted in loss of or decrease in additional, unidentified key factor(s) important for viral entry since susceptibility to HCVpp was significantly recovered by ectopic expression of CD81 but HCVpp infectivity in CD81-expressing Hdo cells remained significantly lower than that in HuH-7 cells (Figure 3E).

While Hdo cells can maintain HCV IRES-dependent translation activity, they have no or little ability to support replication of viral replicons (Figure 4A), suggesting the influence of epigenetic reprogramming on regulatory mechanisms for post-translation steps during HCV replication. Expression levels of known host factors involved in HCV replication such as miR-122, PI4K, VAPs, and SEC14L2 in Hdo cells were comparable to those in HuH-7 cells (Supplementary Figure 4A). However, from our miRNA array and subsequent analyses, miR200a-3p was identified as a novel host factor that is potentially involved in negative regulation of HCV replication. Interestingly, it appears that miR200a-3p expression in cells is dependent on hepatic differentiation stages and its expression levels in mature cells are quite low, e.g., miR200a-3p RNA level in HuH-7 cells was approximately 10-fold lower than that in Hdo cells (Figure 5B) and the level in iPS-derived hepatocytes was 35-, 41-, 116-, and 125-fold lower than that in iPS, iPS-derived definitive endoderm, early hepatic endoderm cells, and hepatoblasts, respectively (Supplementary Figure 5A). It has been reported that several transcription factors rich in undifferentiated cells such as c-Myb, ZEB1/2, and SOX2 are involved in miR200a expression [18]. From an *in silico* miRNA target search, two sites, one in the E2 coding region and the other in the NS4B coding region, were identified as possible target sites (6-mer matched) within the HCV genome. Among human genes, ZFR (NM_016107), RANBP6 (NM_012416), and ZEB2 (NM_001171653), whose roles in HCV replication have not been reported to date, were predicted as targets of miR200a-3p. Studies to evaluate the interaction of these candidate virus- and cell-derived targets with miR200a-3p and their involvement in HCV replication are under way.

We demonstrated that induction of hepatocyte-like cells from Hdo cells led to restoration of susceptibility to HCV replication to some extent (Figure 5A), suggesting that cell characteristics of hepatic differentiation or maturation are advantageous for supporting viral replication. However, HCV replication efficiency in Hdo-derived hepatocyte-like cells was lower than that in HuH-7 cells. Although previous studies showed the support of HCV propagation in hepatocyte-like cells induced from human ES cells or iPS cells, its permissiveness appeared to be lower in these cells compared to HuH-7 cells [4,6,29]. It is generally accepted that currently developed conditions for differentiation induction from stem cells lead to partial, but not complete, hepatic function. It may be likely that loss of expression of unidentified factor(s) important for HCV replication, potentially via epigenetic modification by expression of reprogramming factors, cannot be recovered by cell treatment aimed at differentiation induction.

In conclusion, we developed HuH-7-derived oval-like cells, Hdo cells, in this study. We also identified a novel host factor, miR200a-3p, that is highly expressed in Hdo cells and poorly-differentiated cells and functions as a negative regulator of HCV replication. We demonstrated that epigenetic reprogramming of human hepatoma cells potentially changes their permissivity to HCV. Hdo cells may be a powerful tool to identify cellular determinants for the HCV life cycle, which change their expression during reprogramming or hepatic differentiation.

## MATERIALS AND METHODS

### Plasmids

Subgenomic replicon plasmids pSGR-JFH1/GLuc and pSGR-JFH1/GLuc-GND (HCV genotype 2a), which carry the *Gaussia princeps* luciferase (GLuc) gene as a reporter, were constructed by substitution of GLuc amplified from pGLuc-Basic 2 vector (New England Biolabs, Beverly, MA, USA) into luciferase (Luc) gene of pSGR-JFH1/Luc and pSGR-JFH1/Luc-GND [30,31]. GDD-to-GND mutation in NS5B abolishes RNA polymerase activity. Subgenomic replicon plasmids of the Con1 strain (HCV genotype 1b), pFK-I389Luci/NS3-3’/NK5.1 [32], were kindly provided by Dr. Ralf Bartenschlager (University of Heidelberg, Heidelberg, Germany). pSGR-I389/GLuc was constructed by substitution of GLuc into the Luc gene of pFK-I389Luci/NS3-3’/NK5.1. pSGR-I389/GLuc-GND was constructed by introduction of a mutation in NS5B by PCR using the primers: 5’-CCGTGTTGAGGAGTCAATCTAC-3’(forward) and 5’-GTGTCTAGCTGTCTCCCACG-3’ (reverse). pSGR-JFH1, pHH/SGR-JFH1/GLuc, pHH/SGR-JFH1/GLuc/GND, pcDNA3.1-CD81, pRLucHCVLuc (H77 IRES; HCV genotype 1a), and pRLucEMCVLuc (EMCV IRES) were constructed as previously described [33–36]. pSV40-CLuc was purchased from New England Biolabs. pNL4-3.Luc.R-.E- was obtained from Dr. Niveen Mulholland through the National Institutes of Health AIDS Reagent Program (Bethesda, MD, USA). pNL4-3.NanoLuc.R−.E− was constructed by substitution of NanoLuc (pNL1.1; Promega, Madison, WI, USA) into the Luc gene of pNL4-3.Luc.R-.E-. pmirGLO Dual-Luciferase miRNA Target Expression Vector (Promega) was digested with *Pme*I and *Xba*I and the fragment was recombined with annealed DNA fragments for miRNA target site and its mutant by the In-Fusion HD cloning kit (Takara, Shiga, Japan). Seed sequences are underlined (miR200a-5p: 5’- GTTTAAACGCGGCCG CACATCGTTACCAGACAGTGTTATCTAGA-3’, miR200a-5pmut: 5’ - GTTTAAACGCGGCCG CACATCGTTACCAGAGACTCTTATCTAGA-3’, miR200a-3p: 5’ - GTTTAAACGCGGCCG CTCCAGCACTGTCCGGTAAGATGTCTAGA-3’, miR200a-3pmut: 5’ - TTTAAACGCGGCCG CTCCAGCACTGTCCGCTTACATGTCTAGA-3’). Plasmids containing HBV genomes pUC-HB-Bj and pUC-HB-CAT [37] were gifts from Dr. Masashi Mizokami (National Center for Global Health and Medicine, Tokyo, Japan).

### Cell culture

Human hepatoma HuH-7, Huh7.5.1 (a gift from Dr. Francis Chisari, The Scripps Research Institute, La Jolla, CA, USA), HepG2 cells and human embryonic kidney 293T cells were maintained as previously described [34]. Extrahepatic bile duct carcinoma TFK-1 cells and intrahepatic bile duct carcinoma HuCCT1 cells were maintained in RPMI 1640 medium supplemented with 100 U/ml of penicillin, 100 μg/ml of streptomycin, and 10% fetal bovine serum (FBS). Hdo and human iPS cell line 253G1 [12] were cultured in mTeSR1 (STEMCELL Technologies, Vancouver, Canada) on a CELLstart CTS (Thermo Fisher Scientific, Rockford, IL, USA) pre-coated dish. HepG2, TFK-1, HuCCT1, and 253G1 cells were provided by the RIKEN BioResource Center (RIKEN BRC) through the National Bio-Resource Project of the Ministry of Education, Culture, Sports, Science and Technology, Tokyo, Japan. HuH 5-15 cells (provided by Dr. Ralf Bartenschlager) support replication of the subgenomic HCV replicon derived from the Con1 strain in HuH-7 cells [38].

### Antibodies

For Immunoblotting, mouse monoclonal antibodies against GAPDH (6C5; Santa Cruz Biotechnology, Santa Cruz, CA, USA), α-tubulin (DM1A; Sigma-Aldrich, St. Louis, MO, USA), HBs (5124A; Institute of Immunology, Tokyo, Japan), cytokeratin 8/18 (CK8, NCL-5D3; Progen Biotechnik, Heidelberg, Germany), LDL receptor (C7; Millipore, Billerica, MA, USA), and occludin (OCLN, OC-3F10; Thermo Fisher Scientific) were used. Mouse monoclonal antibodies against AFP (3H8), CK19 (BA17), and EpCAM (VU1D9) were obtained from Cell Signaling Technology (Danvers, MA, USA). Rabbit polyclonal antibodies against albumin (Cell Signaling Technology), SR-BI (Novus Biologicals, Littleton, CO, USA), and claudin-1 (CLDN1; Thermo Fisher Scientific) were used. Rabbit polyclonal antibody against HBc was generated by immunizing rabbits with bacterially-expressed HBc protein. For immunocytochemistry, rabbit polyclonal antibody against β-catenin (Abcam plc, Cambridge, UK), albumin (MP Biomedicals, Santa Ana, CA, USA), CLDN1 (Thermo Fisher Scientific), and LDL receptor (Bethyl Laboratories, Montgomery, TX, USA), mouse monoclonal antibodies against AFP (Cell Signaling Technology), CK8/18 (Santa Cruz Biotechnology), CK19 (Cell Signaling Technology), CD81 (BD Biosciences, San Jose, CA, USA), SR-BI (Cell Signaling Technology), OCLN (Thermo Fisher Scientific), EpCAM (Cell Signaling Technology), and dsRNA (J2; English & Scientific Consulting, Szirak, Hungary), anti-albumin goat polyclonal antibody in Human Albumin ELISA kit (Bethyl Laboratories), and Alexa Fluor 488-conjugated anti-phalloidin (Molecular Probes, eugene, OR, USA) were used.

### Reprogramming of HuH-7 cells

HuH-7 cells were transduced with the retroviral vector encoding *OCT3/4*, *SOX2*, *KLF4*, *LIN28*, and *NANOG* (Human iPS Cell Generation All-in-One Vector; Takara) by the RetroNectin-bound virus infection method [39]. After 6 days, transduced cells were plated onto an STO feeder cell layer. The medium was replaced with Primate ES Cell Medium (Repro CELL, Kanagawa, Japan) containing 4 ng/ml bFGF (Takara) every 2 days. Colonies were observed 40 days after transduction, and colonies morphologically similar to human iPS cells were selected for further cultivation and evaluation.

### Differentiation of hepatocytic and cholangiocytic cells

For induction of hepatic lineage, Hdo cells (1×10^5^ cells/6-well plate) were incubated in hepatic differentiation medium [40]. The hepatic differentiation medium comprised DMEM/F12 (Sigma-Aldrich) supplemented with 10% FBS, nonessential amino acids (Thermo Fisher Scientific), 0.1 μM dexamethasone (Sigma-Aldrich), 1x insulin-transferrin-sodium selenite supplement (Thermo Fisher Scientific), 10 ng/ml oncostatin M (OSM) (R&D Systems, Minneapolis, MN, USA), 10 ng/ml recombinant human HGF (PeproTech, Rocky Hill, NJ, USA) and 10 ng/ml recombinant human FGF-4 (PeproTech).

For induction of cholangiocytic lineage [41], Hdo cells were trypsinized into a single-cell suspension of 5×10^4^ cells/ml in a mixture of buffered, liquefied collagen type I-A (Nitta Gelatin, Tokyo, Japan) and Matrigel Growth Factor Reduced (Becton Dickinson, Franklin Lakes, NJ, USA). Cells in the gel mixture solution were plated onto cell culture inserts (10.5 mm in diameter, 0.4-μm pore size, Becton Dickinson). The gel mixture was allowed to set at 37°C for 2 h, and then cholangiocytic differentiation medium was added on top of and below the insert. The cholangiocyte differentiation medium comprised DMEM/F12 supplemented with 10% FBS, 10 mM nicotinamide (Sigma-Aldrich), 0.1 μM dexamethasone, 1x insulin-transferrin-sodium selenite supplement, 5 ng/ml recombinant human EGF (PeproTech), and recombinant human HGF. Cells were fed every 3 days and grown for 2 weeks until cysts with lumina or tubular structures formed. Before fixation, gels of type I collagen mixed with Matrigel were treated with 0.02% collagenase (Wako, Osaka, Japan) in DMEM/F12 for 30 min at 37°C to increase the accessibility of the cells to antibodies.

For preparation of hepatic differentiated cells from iPS cells, a human iPS cell line, 201B7, was obtained from RIKEN BRC [42]. 201B7 cells were cultured in primate ES cell medium supplemented with 4 ng/ml of recombinant human bFGF (R&D Systems) on a mitomycin C-treated mouse embryonic fibroblast feeder layer. To maintain iPS cells in an undifferentiated state, iPS cells were passaged every 3-4 days at approximately 80% confluency using a StemPro EZpassage tool (Thermo Fisher Scientific) onto a fresh MEF feeder layer. To initiate definitive endoderm cell differentiation, 201B7 cells were stimulated with 100 ng/ml of recombinant human activin A (R&D Systems) in RPMI 1640 medium for 3 days. Harvested definitive endoderm cells were plated on 6-well plates pre-coated with 1 mg/ml EHS gel (Corning, Corning, NY, USA) to process further differentiation steps. Early hepatic endoderm cells were induced by 20 ng/ml of BMP-2 (R&D Systems) and 10 ng/ml of FGF-4 treatment in hepatocyte culture medium (Lonza, Basel, Switzerland), followed by hepatoblast induction, 20 ng/ml HGF treatment for 5 days, and finally stimulated with 20 ng/ml of OSM and 100 nM of dexamethasone to induce hepatocyte maturation.

### Preparation of viral stocks and viral infections

Cell culture-derived infectious HCV particles (HCVcc) were prepared as described previously [31,35]. Pseudotypes were generated by transfection of 293T cells (plated at 1×10^6^ cells/10-cm dish 24 h prior to transfection) with 2.5 μg of pNL4-3.NanoLuc.R−E− plasmid containing the env-defective human immunodeficiency virus type 1 (HIV-1) proviral genome and 2.5 μg of expression plasmid encoding HCV JFH-1 envelope glycoproteins or vesicular stomatitis virus protein G (VSV-G) with Lipofectamine LTX (Invitrogen, Carlsbad CA, USA). The medium (5 ml/dish) was replaced 12 h after transfection. Supernatants containing pseudotypes (HCVpp and VSVpp) were collected 60 h later. The supernatants were stored at -80°C until use. Cells seeded onto 12- or 24-well plates were infected with HCVcc at a multiplicity of infection (MOI) of 0.1-5 or with diluted supernatant containing HCVpp or VSVpp [43].

miR-122-encoding lentivirus was prepared as described [17]. Briefly, lentiviral vectors, pCSII-EF-WT-miR-122 and pCSII-EF-AcGFP, and ViraPower Lentiviral Packaging Mix (Invitrogen) were co-transfected into 293T cells, and the supernatants were recovered at 48 h post-transfection. The lentivirus titer was determined by a Lenti-XTM quantitative reverse transcription-PCR (qRT-PCR) titration kit (Clontech, Mountain View, CA, USA), and the expression levels of miR-122 and AcGFP were determined at 48 h post-inoculation.

JEV were prepared as described [44].

### RNA extraction and qRT-PCR

Total cellular RNAs isolated by TRI reagent (Cosmo Bio, Tokyo, Japan) were transcribed using the SuperScript VILO cDNA Synthesis Kit (Thermo Fisher Scientific). Aliquots of cDNAs were subjected to 45 cycles of PCR amplification. The primer sequences used are shown in Supplementary Table 2. qRT-PCR was performed in the CFX Connect Real-Time System (Bio-Rad, Hercules, CA, USA) using THUNDERBIRD SYBR qPCR mix (Toyobo, Osaka, Japan). Briefly, reversed transcribed cDNAs together with 6 pmol of forward and reverse primers were used for PCR. The thermal cycling conditions comprised 1 min at 95°C, followed by 40 cycles at 95°C for 15 s and 60°C for 30 s. RNA expression data were normalized to that of *GAPDH* using the comparative threshold method (∆∆CT).

### Quantification of HCV RNA

HCV RNA copies were determined using qRT-PCR as described previously [45] with the CFX Connect Real-Time System (Bio-Rad).

### Quantification of HBV DNA

Quantification of HBV DNA was essentially performed as described previously [46]. To quantify particle-associated HBV DNA, culture supernatants collected from transfected cells were treated with PNE solution (8.45% PEG, 0.445 M NaCl, 13 mM EDTA) for 1 h on ice. To remove free nucleic acids, pellets were incubated at 37°C for 1 h with DNase I and RNase. After treatment with proteinase K at 56°C overnight, HBV DNA was isolated by phenol/chloroform extraction and ethanol precipitation. HBV DNA copies were determined by quantitative PCR (qPCR). The primer sequences used are shown in Supplementary Table 2.

### Microarray

mRNAs were labeled with Cy5 mono-reactive dye (GE Healthcare, Little Chalfont, UK) and purified according to the manufacturer's protocol (3DGENE mRNA CyDye label v2, Toray Industries, Tokyo, Japan). The concentration of labeled RNAs was determined by a NanoDrop 1000 spectrophotometer (Thermo Fisher Scientific) before hybridization onto microarray chips (Human Oligo Chip 25k ver. 2.10., Toray Industries). Hybridization and subsequent washing were performed according to the manufacturer's protocol (3DGENE mRNA hybridization v2, Toray Industries). For miRNA analysis, miRNA was labeled, hybridized, and washed according to the manufacturer's protocol (3DGENE miRNA label Hybridization 4plex v1, Toray Industries) using the miRCURY LNA microRNA Array Power Labeling kit (Exiqon, Vedbaek, Denmark) and Human miRNA oligo chip (Human miRNA Chip ver. 16.1.0.0, Toray Industries). The intensity of labeled mRNAs or miRNAs was analyzed with the 3D-Gene Scanner 3000 system with auto gain, auto focus, and auto analysis settings.

### RNA synthesis and electroporation

RNA synthesis was performed as previously described [31]. Briefly, pSGR-JFH1/GLuc, pSGR-JFH1/GLuc-GND, and pSGR-JFH1/Neo were digested with *Xba*I. pSGR-I389/GLuc and pSGR-I389/GLuc-GND were digested with *Sca*I. Digested plasmid DNA fragments were then purified and used as templates for RNA synthesis. HCV RNA was synthesized *in vitro* by a MEGAscript T7 kit (Ambion, Austin, TX, USA). Synthesized RNA was treated with DNase I, followed by acid phenol extraction to remove any remaining template DNA. Trypsinized cells were washed with phosphate-buffered saline (PBS) and resuspended at 1×10^5^ cells/10 μl with BTXpress buffer (BTX, Holliston, MA, USA). One microgram of reporter replicon RNA was then mixed with 10 μl of cell suspension and electroporated by Neon (Thermo Fisher Scientific). The condition of electroporation was at 1,400 V, 20 ms, and 1 pulse. Transfected cells were immediately seeded into 24-well plates. Culture supernatant (20 μl) collected from cells expressing plasmids carrying the GLuc reporter was used to determine GLuc activity by using a BioLux *Gaussia* Luciferase Assay Kit (New England Biolabs).

### miRNA and DNA transfection

miRNAs and non-targeting control miRNA (miR cont) were synthesized by Bonac (Fukuoka, Japan). Antisense locked nucleic acid inhibitors (miRCURY LNA microRNA power inhibitor, Takara) were used for inhibition of miRNA. Cells were transfected with 50 pmol miRNAs or miRNA inhibitors together with Xfect microRNA Transfection Reagent (Takara) or ScreenFect A (Wako). For DNA transfection, Lipofectamine LTX or Lipofectamine 3000 reagent (Thermo Fisher Scientific) was used.

### Quantification of miRNA

Total RNA was isolated using TRI reagent (Cosmo Bio) and cDNA was synthesized from 10 ng of total RNA using the TaqMan microRNA Reverse Transcription Kit with an miRNA-specific RT primer from the TaqMan microRNA Assay (Applied Biosystems, Foster City, CA, USA). miRNA levels were measured using the miRNA-specific TaqMan probe provided in the TaqMan microRNA assays and the THUNDERBIRD probe qPCR mix (Toyobo). Expression of miR-122 and miR-200a were normalized to that of Z30 snoRNA (SNORD7) and fold change was determined by the ∆∆CT method.

### Immunoblotting

Immunoblotting was performed essentially as described previously [47]. Cell lysates with 1% NP-40, 0.1% SDS, 1% sodium deoxycholate, 25 mM Tris-HCl, pH 7.6, 150 mM NaCl, 1 mM EDTA, and protease inhibitor cocktail (Roche Diagnostics, Basel, Switzerland), were separated by SDS-PAGE and transferred onto polyvinylidene difluoride membranes. After blocking for 3 h, membranes were incubated with primary antibody overnight at 4°C. After washing, membranes were incubated with horseradish peroxidase-conjugated secondary antibody (Cell Signaling Technology) for 1 h. Antigen-antibody complexes were detected using ECL Prime Western Blotting Detection Reagent (GE Healthcare).

### Cell staining and indirect immunofluorescence

Alkaline phosphatase (ALP) staining was performed using the Leukocyte Alkaline Phosphatase Kit (85L3R; Sigma-Aldrich) according to the manufacturer's instructions. Periodic acid-Schiff (PAS) staining was performed using the Periodic acid-Schiff Kit (Sigma-Aldrich) according to the manufacturer's instructions. For indirect immunofluorescence, cells grown on a glass bottom plate were fixed with 4% paraformaldehyde (Wako) in PBS for 30 min at 4°C. For CD81, OCLN, CLDN1, EpCAM, and CK19 staining, cold acetone was used for fixation for 10 min at -20°C. For SR-BI staining, cold acetone/ethanol was used for fixation for 10 min at -20°C. Cells were permeabilized in 0.5% Triton X-100 in PBS for 10 min at room temperature, followed by blocking with 1% bovine serum albumin. Immunocytochemistry was performed with a primary antibody overnight at 4°C, followed by incubation with Alexa Fluor 488 goat anti-mouse IgG, Alexa Fluor 488 goat anti-rabbit IgG, or Alexa Fluor 594 goat anti-mouse IgM (Invitrogen) for 1 h at room temperature. Double-stranded DNA was stained with Hoechst 33342 (Dojin, Tokyo, Japan) or DAPI (Sigma-Aldrich). Confocal images were acquired with FV1000-D (Olympus, Tokyo, Japan). Cell morphology was analyzed by three-dimensional low-coherent quantitative phase microscopy (Hamamatsu Photonics, Shizuoka, Japan) [48].

### Flow cytometric analysis

After 2 days of transfection with pcDNA3.1 or pcDNA-CD81, subconfluent cells seeded in 60-mm dishes were harvested and incubated with mouse anti-CD81 antibody (JS-81; Pharmingen) or mouse IgG1κ (eBioscience, San Diego, CA, USA) for 30 min at 4°C and washed with PBS. Cells were then incubated with a PE-conjugated anti-mouse IgG1 secondary antibody (eBioscience) at 1:100 for 30 min at 4°C, washed repeatedly, and resuspended in PBS containing 1% (vol/vol) formaldehyde. Flow cytometric analyses were performed using Epics XL (Beckman Coulter, Fullerton, CA).

### Luciferase assay

Cells seeded in a 24-well plate at a density of 5×10^4^ cells/well were transiently transfected with reporter vectors. After 2 days, cells were harvested and lysed with 150 μl of passive lysis buffer (Promega). Luciferase activities were measured using the Dual-Luciferase Reporter Assay System (Promega), Nano-Glo Luciferase Assay System (Promega), or BioLux Dual Luciferase Starter Kit (New England Biolabs) and the Infinite M200 Pro system (TECAN, Kanagawa, Japan). Luciferase activities were normalized to *Renilla* luciferase activity.

### Cell viability assay

Cell viability was determined using the CellTiter-Glo Luminescent Cell Viability Assay (Promega) according to the manufacturer's instructions.

### Colony formation assay

Huh7.5.1 cells were transfected with 50 pmol miRNAs or miRNA inhibitors together with Xfect microRNA Transfection Reagent. After 1 day, *in vitro*-transcribed SGR-JFH1/Neo RNA was electroporated into cells using Neon and plated on DMEM containing 10% FBS. The culture medium was replaced with fresh DMEM containing 10% FBS and 500 μg/ml G418 at 48 h post-electroporation. Transfection with miRNAs or miRNA inhibitors was repeated at 2 and 9 days post-electroporation. Cells were fixed with 4% paraformaldehyde in PBS and stained with crystal violet at 12 days post-electroporation.

### Statistical analysis

Values are expressed as means of triplicate experiments with the standard error of the mean (SEM). In most analyses, statistical significance was calculated using the unpaired Student's *t*-test. Differences were considered significant at *p* < 0.05.

## SUPPLEMENTARY MATERIALS FIGURES AND TABLES







## Abbreviations

AAT: alpha 1 Antitrypsin
AFP: alpha-Fetoprotein
ALB: albumin
ATP: adenosine triphosphate
BMP: bone morphogenetic protein
CD81: cluster of differentiation 81
CK19: cytokeratin 19
Cluc: *Cypridina* Luciferase
CYP3A4: cytochrome P450 3A4
DLK1: delta like non-canonical Notch ligand 1
GAPDH: glyceraldehyde 3-phosphate dehydrogenase
Gluc: *Gaussia* Luciferase
EGFR: epidermal growth factor receptor
EMCV: encephalomyocarditis virus
EpCAM: epithelial cell adhesion molecule
ES: embryonic stem
FACS: fluorescence activated cell sorting
FGF: fibroblast growth factor
HNF4A: hepatocyte nuclear factor 4 alpha
IRES: internal ribosome entry site
KLF4: Kruppel like factor 4
LDL: low-density lipoprotein
miRNA: micro-RNA
OCLN: Occludin
OCT: octamer-binding transcription factor
REX1: reduced-expression 1
SGR: subgenomic replicon
SOX2: sex determining region Y-box 2
SR-BI: scavenger receptor class B member
SSEA: stage-specific embryonic antigen-1
TAT: tyrosine aminotransferase
TTR: transthyretin.
