# Supplementary material for: Development of hepatoma-derived, bidirectional oval-like cells as a model to study host interactions with hepatitis C virus during differentiation
Source: Oncotarget. 2017 Jul 8;8(33):53899–915. doi: 10.18632/oncotarget.19108 (PMC5589550; doi:10.18632/oncotarget.19108)
Supplement: Supplementary file 1 [file oncotarget-08-53899-s001.pdf]

Development of hepatoma-derived, bidirectional oval-like cells as a model to study host interactions with hepatitis C virus during differentiation

SUPPLEMENTARY MATERIALS

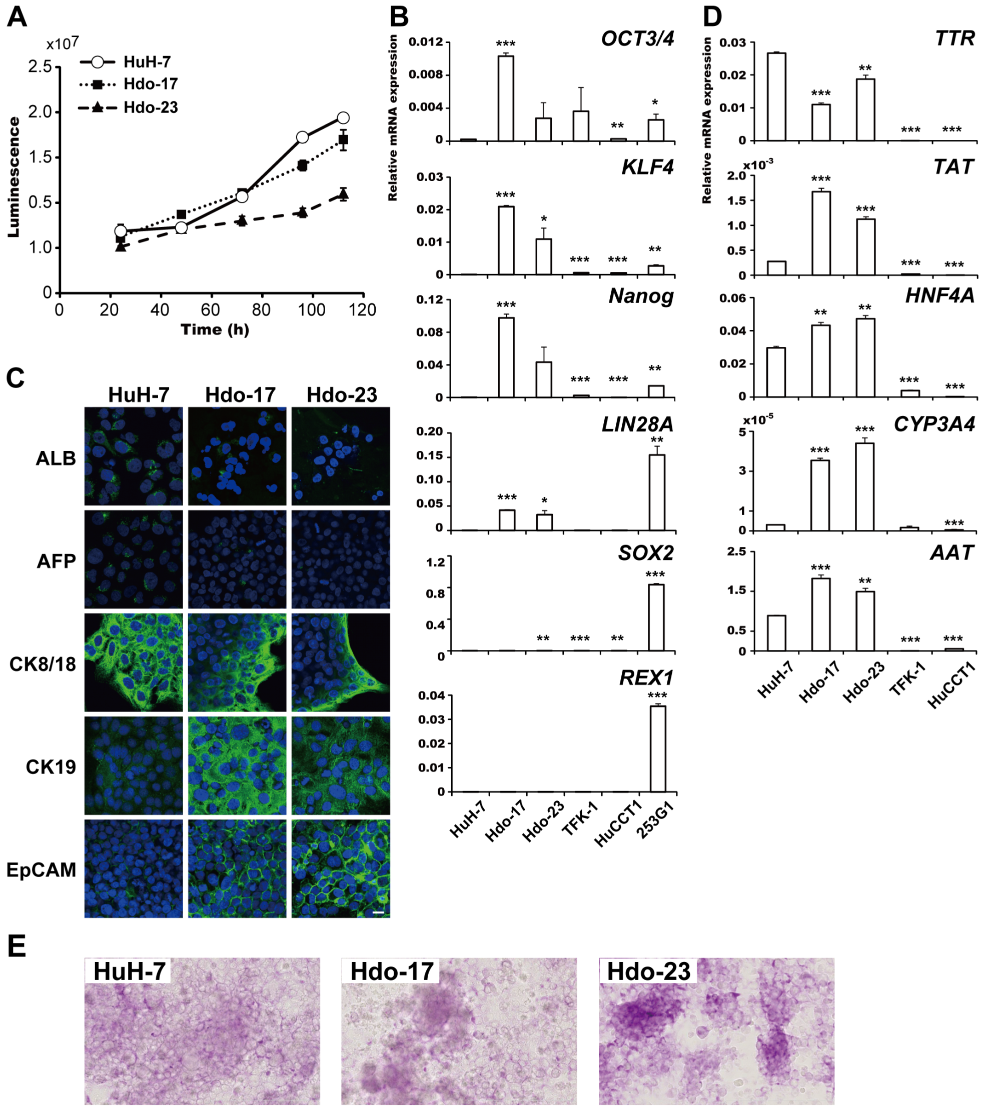

Supplementary Figure 1: (A) HuH-7 (white circles), Hdo-17 (black squares), and Hdo-23 (black triangles) cells were plated in 24-well plates at 1×105 cells/well. ATP concentrations in cells were measured by the CellTiter-Glo Luminescent Cell Viability Assay at the defined time points. (**B**) At 5 days after passage, total RNA was extracted from HuH-7, Hdo-17, Hdo-23, TFK-1, HuCCT1, and 253G1 cells. Expression of pluripotency markers was measured by qRT-PCR. (**C**) At 5 days after passage, expression of liver markers in HuH-7 (left), Hdo-17 (middle), and Hdo-23 (right) cells was examined by immunostaining (green) together with DAPI (blue). Bar indicates 20 µm. (**D**) At 5 days after passage, total RNA was extracted from HuH-7, Hdo-17, Hdo-23, TFK-1, and HuCCT1 cells. Expression of liver markers was measured by qRT-PCR. (E) Expression of mucopolysaccharides in HuH-7, Hdo-17, and Hdo-23 cells was determined by staining with the Periodic acid-Schiff Kit at 1 day after passage (6× objective). All assays were performed in triplicate. (A), (B), and (D) Results are presented as means ± SEM (n=3). (B) and (D) Data were normalized to the expression of GAPDH mRNA. Statistically significant differences compared with HuH-7 cells are shown. *p < 0.05, **p < 0.01, ***p < 0.001, Student's t-test.

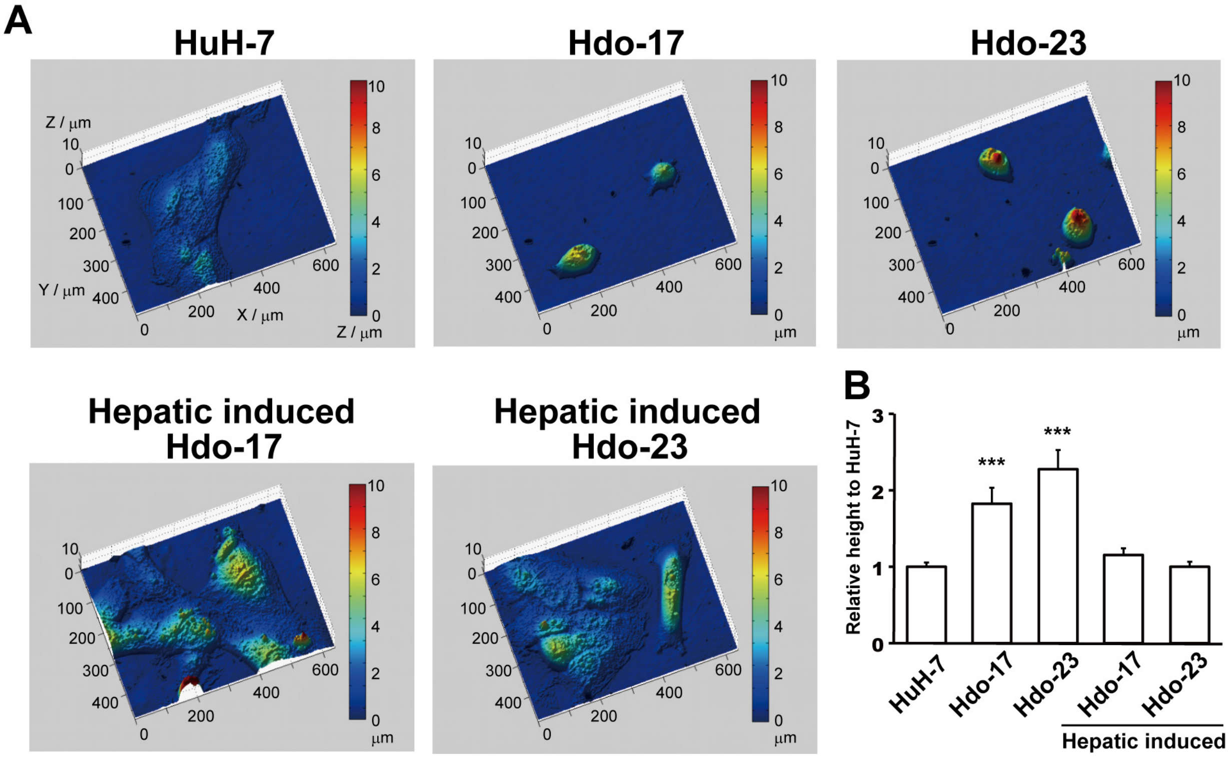

Supplementary Figure 2: (A) HuH-7, Hdo-17, Hdo-23, and respective hepatic induction cells were plated on glass bottom 35-mm dishes at 0.5×105 cells. At 1 day after plating, cell morphology was analyzed by a quantitative phase microscope (3D LC-QPM). 3D topographic optical thickness maps of a region measuring 650 µm (horizontal) × 475 µm (vertical) × 10 µm (height) are shown. (**B**) The mean maximum height of each cell (n=20) was calculated from the images. Relative height to HuH-7 is indicated. Results are presented as means ± SEM (n=20). Statistically significant differences compared with HuH-7 cells are shown. $^*p < 0.05$, $^{**}p < 0.01$, $^{***}p < 0.001$, Student's t-test.

A

	Hdo-17	Hdo-23
OCLN	1.97	1.69
CD81	0.08	0.10
CLDN1	1.04	1.16
SR-BI	0.94	0.98
LDLR	0.49	0.66
EGFR	0.94	1.44
EPHA2	0.61	0.48
CD229	0.69	0.68
NPC1L1	0.61	0.64
PI4KA	0.70	0.86
VLDLR	1.17	1.04

0 1 2

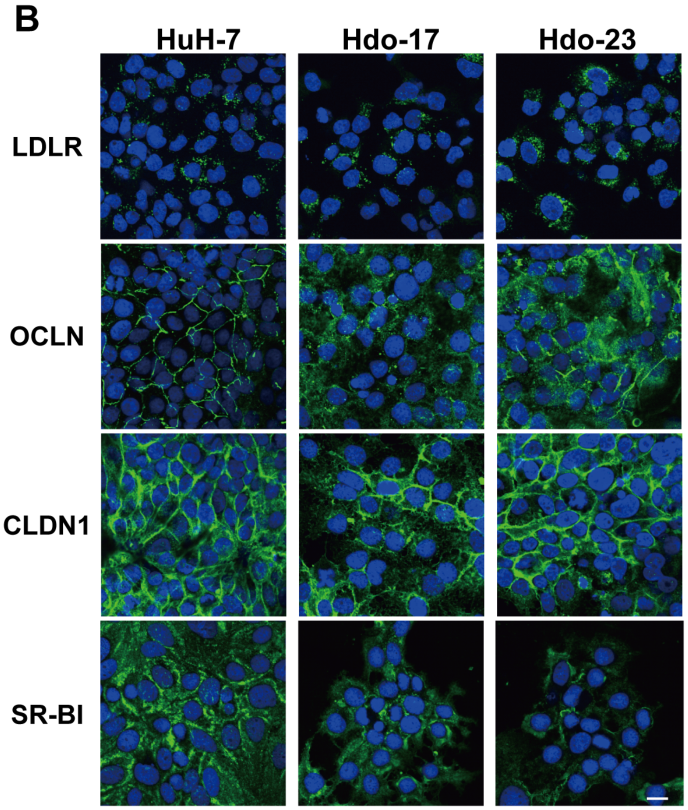

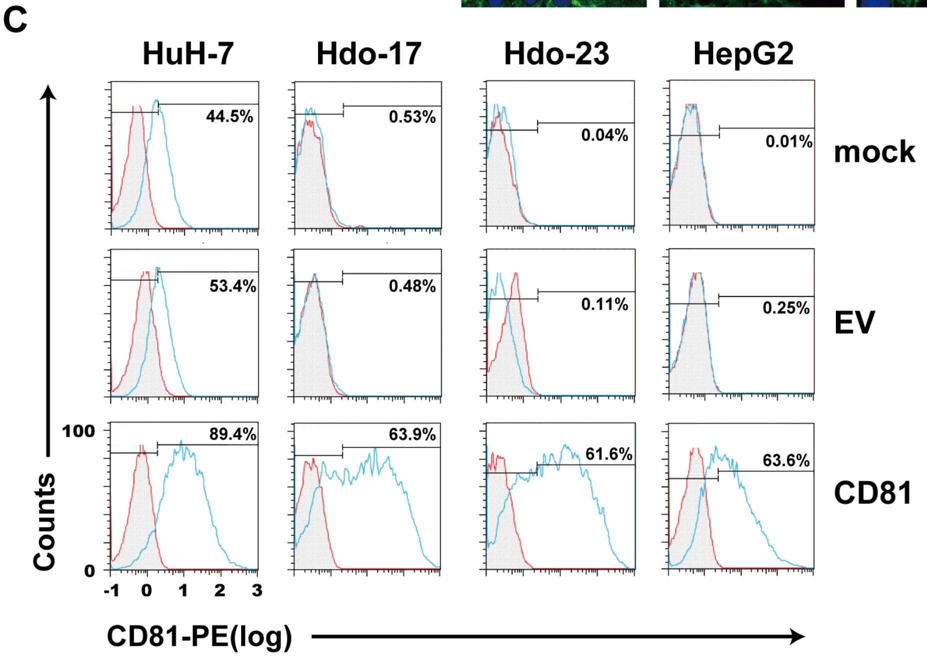

Supplementary Figure 3: (A) Microarray heat map of mRNA expression levels of possible key factors for HCV entry in Hdo-17 or Hdo-23 cells versus HuH-7 cells. Two independent RNA samples were processed, and the numbers shown represent the ratios of mean values in Hdo-17 or Hdo-23 cells against those in HuH-7 cells. The conventional color spectrum with blue representing downregulation and red representing upregulation was adopted. (**B**) At 5 days after passage, expression of HCV entry factors in HuH-7 (left), Hdo-17 (middle), and Hdo-23 (right) cells was examined by immunostaining (green) together with DAPI (blue). Bar indicates 20 μm. (**C**) Huh-7, Hdo-17, Hdo-23, and HepG2 cells were transfected with pcDNA3.1 (EV) or pcDNA-CD81 (CD81). Expression of cell surface CD81 was examined by staining with anti-CD81 antibody and control IgG, followed by incubation with phycoerythrin (PE)-conjugated anti-mouse IgG1 antibody. Flow cytometric analysis was performed by Epics XL. The x- and y-axes show fluorescence intensity and relative number of stained cells, respectively.

A

Gene	Hdo-17	Hdo-23
ACTN1	1.10	1.25
CHEK2	0.34	0.76
CKB	0.71	2.95
CARD14	2.03	1.49
DDX3X	3.86	2.54
DDX5	1.89	1.51
DDX6	0.95	1.14
DGAT1	0.88	0.93
DZIP3	0.92	0.98
FBXL2	1.16	1.31
FKBP8	1.21	1.21
FUBP1	1.20	0.97
HNRNPA1P2	0.57	0.93
HNRNPC	0.83	0.80
HNRNPL	0.87	0.78
HSPB1	0.72	0.87
HSP90AA2	0.98	1.64
ILF3	1.48	1.27
NCL	1.44	1.50
NIPSNAP1	0.70	0.70
NPC1L1	0.82	0.64
PARD6A	1.19	1.10
PI4KA	0.73	0.86
PKM2	1.27	1.56
PPIA	1.32	1.48
PPIB	1.98	1.74
PPIC	2.03	1.67
PTBP1	1.08	1.11
PTPLAD1	0.97	0.95
RAB5A	1.61	1.29
RAF1	1.28	1.09
SEC14L2	1.31	1.74
Septin6	1.33	1.16
SYNCRIP	1.37	1.25
SSB	2.32	1.78
TBC1D20	1.09	0.90
TRAF2	0.93	1.11
TRiC/CCT	1.14	1.13
VAPA	1.20	0.86
VAPB	0.90	0.85

B

miRNA	Hdo-17	Hdo-23
hsa-miR-141	36.14	21.09
hsa-miR-145	6.20	1.36
hsa-miR-146a	24.79	17.00
hsa-miR-146b-5p	6.84	4.80
hsa-miR-152	0.31	0.26
hsa-miR-200a	11.69	7.73
hsa-miR-200b	8.65	5.44
hsa-miR-200c	32.33	22.12
hsa-miR-451	6.57	4.03
hsa-miR-483-3p	0.33	0.43
hsa-miR-711	3.61	6.11
hsa-miR-1274a	0.48	0.35
hsa-miR-1290	3.99	5.41
hsa-miR-4294	6.59	11.27
hsa-miR-122	0.83	0.90

Supplementary Figure 4: (A) mRNA expression levels of genes associated with HCV replication in Hdo-17, Hdo-23, and HuH-7 cells were examined by microarray chips (Human Oligo Chip 25k ver. 2.10.). Two independent RNA samples were processed. The value indicates the ratio of RNA expression level in Hdo cells compared to that in HuH-7 cells. (**B**) miRNA expression levels in Hdo-17 or Hdo-23 cells and HuH-7 cells were examined by microarray chips (Human miRNA Chip ver. 16.1.0.0). Two independent RNA samples were processed. The value indicates the ratio of RNA expression levels in Hdo cells compared to that in HuH-7 cells. Expression of miR-122 in Hdo-17 and -23 cells was also compared to that in HuH-7 cells, as indicated at the bottom.

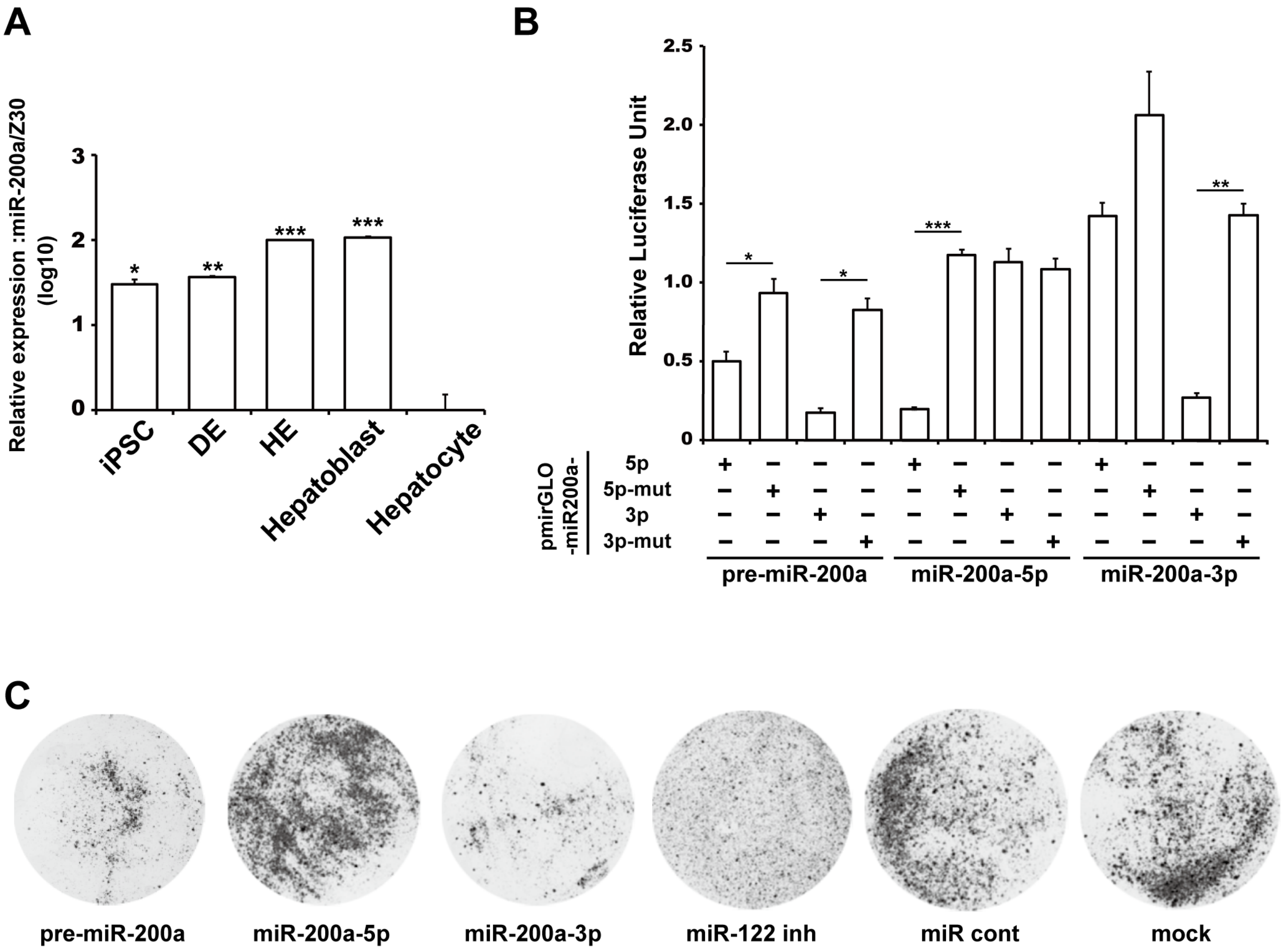

Supplementary Figure 5: (A) iPS cells (iPSC, 201B7) were differentiated into hepatocytes. Expression of miR-200a in differentiated stages (definitive endoderm (DE), early hepatic endoderm (HE), hepatoblast, and hepatocyte) was examined by qRT-PCR. Expression of miR-200a was normalized to that of Z30 snoRNA (SNORD7), and relative expression levels in hepatocyte are shown. (**B**) HuH-7 cells were transfected with pre-miR-200a, mature miR-200a-5p, or miR-200a-3p. At 1 day after transfection, cells were transfected with pmirGLO Dual-Luciferase miRNA Target Expression Vector comprising the predicted miR-200a target sequence and its mutant (mut). The relative firefly luciferase activity normalized to Renilla luciferase activity was measured 1 day after pmirGLO transfection. (**C**) Colony formation of JFH-1 HCV subgenomic RNA replicon. Transcribed RNAs were transfected into HuH-7 cells, and cells were cultured with G418 for 12 days before staining with crystal violet. (A) and (B) Results are presented as means ± SEM (n=3). *$p < 0.05$, **$p < 0.01$, ***$p < 0.001$, Student's t-test. (A)-(C) Assays were performed in triplicate.

For Supplementary Tables see in Supplementary Files
