# Supplementary material for: Development of hepatoma-derived, bidirectional oval-like cells as a model to study host interactions with hepatitis C virus during differentiation
Source: Oncotarget. 2017 Jul 8;8(33):53899–915. doi: 10.18632/oncotarget.19108 (PMC5589550; doi:10.18632/oncotarget.19108)
Supplement: Supplementary file 3 [file oncotarget-08-53899-s003.docx]

**Table S2: Primer sequences used for quantitative RT-PCR.**

Gene name Forward (5’ to 3’) Reverse (5’ to 3’)

ALB AATGTTGCCAAGCTGCTGA CTTCCCTTCATCCCGAAGTT

AFP GGCCTGTTGGAGAAATGCT CAAAGCAGCACGAGTTTTTG

EpCAM CCATGTGCTGGTGTGTGAA TGTGTTTTAGTTCAATGATGATCCA

CK19 GTCATGGCCGAGCAGAAC CCGGTTCAATTCTTCAGTCC

DLK1 GACGGGGAGCTCTGTGATAG TCATAGAGGCCATCGTCCA

SRBI CATCAAGCAGCAGGTCCTTA CGGAGAGATAGAAGGGGATAGG

LDLR AGGACGGCTACAGCTACCC CTCCAGGCAGATGTTCACG

CLDN1 GCGCGATATTTCTTCTTGCAGG TTCGTACCTGGCATTGACTGG

OCLN AGGAACCGAGAGCCAGGT GGATGAGCAATGCCCTTTAG

CD81 TCGTCTTCAATTTCGTCTTCTG CTCCCAGCTCCAGATACAGG

NPC1L1 TATGGTCGCCCGAAGCA TGCGGTTGTTCTGGAAATACTG

EGFR TTCCTCCCAGTGCCTGAA GGGTTCAGAGGCTGATTGTG

OCT3/4 AAGCGATCAAGCAGCGACTAT GGAAAGGGACCGAGGAGTACA

KLF4 GGCACTACCGTAAACACACG CTGGCAGTGTGGGTCATATC

Nanog CAAAGGCAAACAACCCACTT TCTGCTGGAGGCTGAGGTAT

LIN28A GAAGCGCAGATCAAAAGGAG GCTGATGCTCTGGCAGAAGT

SOX2 GGGGGAATGGACCTTGTATAG GCAAAGCTCCTACCGTACCA

REX1 GGCCTTCACTCTAGTAGTGCTCA CTCCAGGCAGTAGTGATCTGAGT

TTR GCCGTGCATGTGTTCAGA GCTCTCCAGACTCACTGGTTTT

TAT GATGTCCCCATCCTGTCCT CTTCACCAGCCCATCTCG

HNF4A CAGCACTCGAAGGTCAAGCTA ACGGGGGAGGTGATCTGT

CYP3A4 GATGGCTCTCATCCCAGACTT AGTCCATGTGAATGGGTTCC

AAT GCACCTGGAAAATGAACTCAC GGGTAAATGTAAGCTGGCAGA

HBV TCCCTCGCCTCGCAGACG GTTTCCCACCTTATGAGTC

EGFR TTCCTCCCAGTGCCTGAA GGGTTCAGAGGCTGATTGTG

GAPDH AACAGCCTCAAGATCATCAGC GGATGATGTTCTGGAGAGCC
